# Intramural leoimyoma without endometrial cavity distortion may negatively affect the ICSI - ET outcome

**DOI:** 10.1186/1477-7827-11-102

**Published:** 2013-10-29

**Authors:** Suleyman Guven, Cavit Kart, Mesut A Unsal, Ersan Odaci

**Affiliations:** 1 Department of Obstetrics and Gynecology, Farabi ART Center, Karadeniz Technical University Faculty of Medicine, Trabzon, Turkey

**Keywords:** ICSI-outcome, Infertility, Leiomyoma, Pregnancy rate, Uterine cavity

## Abstract

**Background:**

To assess the impact of intramural fibroids on the intracytoplasmic sperm injection-embryo transfer (ICSI-ET) cycle outcome, when there is no compression of the endometrial cavity.

**Methods:**

In this retrospective, matched control study, the ICSI-ET outcome of sixty-two patients (Group I) with intramural fibroid (mean diameter <7 cm) and normal endometrial cavity demonstrated by office hysteroscopy was compared with matched-control group of patients (n = 301) with no fibroid (Group II). The diagnosis of fibroids was done by transvaginal ultrasonography.

**Results:**

The mean age in fibroid group was 32.66 +/- 5.30 while this figure was 32.95 +/- 3.98 in control group. The clinical pregnancy rate was significantly lower in the fibroid group although fibroids not distorting the uterine cavity (25.8% vs. 39.9%, p = 0.04). In fibroid group the implantation rate was significantly lower than control group (20.97 +/- 37.93 vs.32.89 +/- 43.18%, p = 0.04). However, spontaneous abortion rate was higher in fibroid group but it did not reach the significant level (12.5% vs. 9.2%, p > 0.05).

**Conclusion:**

Women having intramural leiomyomas not encroaching on the uterine cavity have unfavorable ICSI/ET outcomes comparable to those of women without such leiomyomas. Therefore, myomectomy may be a good option for such patients with intramural fibroids even they do not have any endometrial distortion.

## Background

Uterine fibroids affect 20–50% of women of reproductive age [1]. They contribute to a variety of clinical problems, including infertility, recurrent pregnancy loss, menorrhagia, and pelvic pressure and fullness, as well as complications of pregnancy. It was estimated that leiomyomata are the sole cause of infertility in only 2–3% of cases when all other factors have been ruled out [2].

Athough the exact mechanism of infertility in women with myoma is unknown, the theories of pathogenic mechanisms include mechanical blockage of tubal ostia impairing sperm or embryo transport through the fallopian tubes, abnormal vascularization, abnormal endometrial development, chronic intracavitary inflammation, an abnormal endocrine milieu, and increased uterine contractility [3].

There is a controversy on the impact of uterine leiomyomata on the outcome of assisted reproductive technologies (ARTs). According to recent review, evidence supports the concept that submucosal fibroids and intramural fibroids that distort the endometrial cavity impair in vitro fertilization (IVF) outcomes. It remains unclear, however, whether intramural and subserosal fibroids, in the setting of a normal endometrial cavity, have similar effects [4].

In some studies it was shown that pregnancy and implantation rates were significantly lower in women with intramural fibroids undergoing IVF/ICSI compared with age-matched controls [5-9]. On the contrary, it other studies, it was reported that IVF/ICSI outcome was not affected by the presence of intramural or subserosal fibroids [10-16].

The purpose of this study was to assess the impact of intramural fibroids with the diameter of <7 cm that do not distort the endometrial cavity on the intracytoplasmic sperm injection-embryo transfer (ICSI-ET) cycle outcome in terms of pregnancy (PR) and abortion rates (AR).

## Methods

### Patients and study design

In this retrospective age matched, controlled study the records of women with uterine fibroids who met the criteria detailed below from a current assisted reproductive technology unit of tertiary health center from July 2009 to June 2013 were reviewed. Institutional review board (Karadeniz Technical University, Faculty of Medicine Clinical Research Ethic commitee) approval was obtained for this retrospective study.

The following inclusion criteria were used: 62 primary infertile women with small uterine intramural (a fibroid which does not distort the uterine cavity and with <50% of its protruding into the serosal surface of the uterus), fundal leiomyomas (mean diameter < 7 cm, as described below) discovered on initial routine screening transvaginal sonography (TVUS) performed in preparation for ICSI-ET and with normal endometrial cavity demonstrated by office hysteroscopy (fibroid group) were retrospectively matched by age and number of collected oocytes with 301 patients at the same period of treatment (same age and same number of collected oocytes) who did not demonstrate fibroids anywhere in the uterus (control group). Only the first cycle of these patients were included.

Women with subserosal, subserosal plus intramural or submucosal, and corpus or cervical or intracavitary leiomyoma were not included in this study. Only women with single intramural fibroid with diameter of <7 cm were included. The patients with multiple fibroids and myoma with the diameter ≥7.0 cm were excluded from the study. All patients had >10 mm distance of fibroids from the cavity. Both the fibroid and control groups had no history of prior myomectomy. All patients in control group were evaluated with hysterosaphingography or sonohysterography for possible endometrial pathologies (polyp, septum, synechia, or etc.). All patients in fibroid and control group had male factor or tubal factor infertility.

### Uterine fibroid diagnosis

Diagnosis of uterine intramural fibroids was done by TVUS performed with a multifrequency endovaginal transducer (Logic p5 Sonography, GE Healthcare, USA). The dimension of each fibroid was determined from the mean value (cm) of the two largest diameters.

### Defining leiomyomas that do not distort the uterine cavity

A woman with leomyoma “no compression of the endometrial cavity” was defined as a patient in whom the endometrium–myometrium transition was clearly seen as a line without distortion of its contours by the presence of the fibroids in both TVUS sagittal and transverse multiple sections of the uterus. Also, findings on an office hysteroscopy before in ICSI-ET cycle were reported as normal for all patients with leiomyoma. All the office hysteroscopy procedures were performed by one of the author within four months of ICSI-ET cycle.

### Ovarian stimulation and patients procedures

All patients were stimulated with luteal phase long protocol. Leuprolide acetate (Lucrin, Abbot, Istanbul, Turkey) was started at the luteal phase. Recombinant-FSH (Gonal F, Serono, Istanbul, Turkey) was administered in a step-down protocol and starting with a 225 IU/day after step down regulation confirmation; and after six days the dose was adjusted according to the ovarian response.

Recombinant human chorionic gonadotropin (250 mcgr SC, Ovitrelle, Serono, Istanbul, Turkey) was administered when at least two or three follicles reached a mean diameter of 17 mm and the serum estradiol concentration was >500 pg/mL. Transvaginal oocyte retrieval was scheduled 36 hours after HCG injection. ICSI was routinely performed for all M II oocytes as our clinical policy.

The embryos that had seven or more cells, symmetric blastomeres, and <10% cytoplasmic fragmentation on day 3 post aspiration were scored as good embryos. Only good quality embryos (single embryo for women age <35 years, two embryos for women age ≥35 years) were transferred. Embryo transfers were performed 72 hours after oocyte retrieval with a Wallace catheter (Sims Portex Ltd., Hythe, United Kingdom) under ultrasound guidance. Luteal support was performed with micronized P 800 mg/day vaginally (Crinone 8% vaginal gell, Serono, Istanbul, Turkey), starting on the day of oocyte retrieval.

### Definitions

Clinical pregnancy was defined as presence of a gestational sac with accompanying fetal heart beat under ultrasound 4 weeks after embryo transfer. Implantation rate was defined as the number of gestational sacs observed on ultrasound compared with the number of embryos transferred.

### Statistics

The ×^2^, Fisher exact test, and *t* test were used for statistical analysis, which was performed using the SPSS software, version 13.0 for Windows (SPSS Inc., Chicago, USA). Statistical significance was set at *P* <0.05.

This retrospective study was conducted without funding sources susceptible of generating any conflict of interest.

## Results

A comparison of the no fibroid and fibroid groups with regards to patients’ clinical and laboratory characteristics are given in Table 1. Since the patients were matched for age and numbers oocyte retrieved there were no significant differences between the 2 groups (32.66 ± 5.3 years vs. 32.95 ± 3.98 years, NS, independent sample *t*-test; and 9.55 ± 4.34 oocytes vs. 9.11 ± 5.13 oocytes, NS, independent sample *t*-test).

**Table 1:** Comparison of clinical and laboratory characteristics in the fibroid and control groups

**Characteristics**	**Fibroid group**	**Control group**	***P*** **value**
	**(n = 62)**	**(n = 301)**	
*Age (yr.)*	32.95 ± 3.98	32.66 ± 5.3	0.683
*Duration of infertility (yr.)*	6.43 ± 6.03	5.63 ± 4.51	0.238
*Baseline FSH (IU/l)*	7.80 ± 3.20	7.29 ± 3.48	0.417
*Total antral follicle count (no.)*	10.52 ± 3.42	11.20 ± 4.76	0.289
*Cause of infertility (%)*			
*Male factor (%)*	53 (85.5%)	249 (82.7%)	0.375
*Tubal factor (%)*	9 (14.5%)	52 (14.5%)	
*No. of oocyte retrieved (no.)*	9.11 ± 5.13	9.55 ± 4.34	0.480
*No. of MII oocyte retrieved (no.)*	6.89 ± 4.29	6.80 ± 3.54	0.871
*No. of embryo transferred (no.)*	1.39 ± 0.49	1.40 ± 0.49	0.866

Values are given as mean ± SD or number of patients (percentage) unless otherwise indicated.

Because only the women with tubal or male factor infertility were included, male factor contributed to the etiology of infertility for most cases in both fibroid and control groups (85.5% vs. 82.7%, respectively, NS, ×^2^ test). Tubal factor was responsible for 14.5% of infertility in the fibroid group and 17.3% in the control group (NS, ×^2^ test).

A comparison of ICSI-ET outcome by the presence of fibrod with the mean diameter 49.61 ± 12.32 cm is given in Table 2. The clinical pregnancy rate (PR) was significantly higher in the control than in the fibroid group (39.9% vs. 25.8%, respectively, p = 0.04, ×^2^ test). In fibroid group the implantation rate was significantly lower than control group (20.97 ± 37.93 vs.32.89 ± 43.18%, p = 0.04). The odds ratio for clinical pregnancy was 0.525 (95% confidence interval 0.284 – 0.969, p = 0.039). However, the first trimester spontaneous abortion rates (AR) for clinical pregnancies was 9.2% (2/16) for fibroid and 12.5% (11/120) for controls (p < 0.05, ×^2^ test). Furthermore the live birth rates were not statistically significantly different in both groups (30.9% (93/301) for no fibroid group, 17.7% (11/62) for fibroid group, p > 0.05, ×^2^ test).

**Table 2:** Comparison of cycle outcome characteristics in the fibroid and control groups

**Characteristics**	**Fibroid group**	**Control group**	***P *** **value**
	**(n = 62)**	**(n = 301)**	
*Fertilization rate (%)*	77.35%	74.72%	0.352
*Implantation rate (%)*	20.97%	32.89%	**0.044**
*Clinical pregnancy rate (%)*	16 (25.8%)	120 (39.9%)	**0.044**
*Spontaneous abortion rate (%)*	2 (12.5%)	11 (9.2%)	0.651
*Live birth rate (%)*	11 (17.7%)	93 (30.9%)	0.086

Values are given as number of patients and/or percentage.

## Discussion

The aim of the current study was to determine whether the presence of intramural fibroids with the diameter of <7 cm has an influence on the PR and AR after ICSI-ET treatment. In this age matched case control study, it was found that the clinical PR per transfer was significantly lower in the group of patients with intramural fibroids, even when there was no deformation of the uterine cavity. However the AR was not significantly affected by the presence of intramural fibroids.

Although many studies have investigated the impact of intramural and subserosal fibroids on IVF outcomes, the effect of intramural fibroids not distorting intrauterine cavity is controversial issue. One recent prospective study results [17] are in agreement with our study. This study compared 112 women with intramural fibroids less than 5 cm in size with 322 women without fibroids. The pregnancy, ongoing pregnancy and live birth rates in the study group were 23.6, 18.8 and 14.8% compared with 32.9, 28.5 and 24% in the control group, respectively (P < 0.05) [17]. Recent meta-analysis also suggested that the presence of non-cavity-distorting intramural fibroids were associated with lower clinical pregnany and live birth rates in women undergoing IVF treatment [18].

A recent prospective study results [15] were also well correlated with our study, although the difference has not been reached statistically significant value. This study compared 61 patients with intramural fibroids less than 5 cm with 61 matched controls. All participants had normal endometrial cavities on hysterosalpingogram, the average fibroid size was 1.5 cm. Compared with the controls, the fibroid group had lower implantation rates (13.6% versus 20.2%), lower pregnancy rates (34.4% versus 47.5%), and higher miscarriage rates (33.3% versus 20.7%), which highlighted the potential negative impact of small intramural fibroids on IVF outcomes [15].

**Figure 1:**
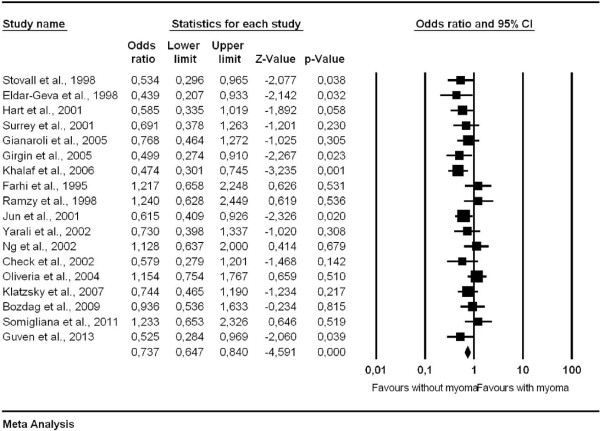
Outcomes, meta-analysis results of all reported studies on pregnancy rates in women with intramural fibroids and in a control group (without myoma).

A recent systematic literature review concerning the effect of fibroids on fertility in patients undergoing ART evaluated six articles and concluded that there was a significant negative impact on implantation rate in the intramural myoma groups vs. the control groups (16.4 vs. 27.7% OR 0.62 (0.48-0.8)). The pregnancy and delivery rates per transfer cycle were also significantly lower in myoma groups. It was concluded that a surgical approach in patients with a history of previous failed attempts with intramural myomata >2 cm in diameter is warranted if all ather factors have been evaluated [9]. Morover, Kolankaya et al. also suggested that there is no consensus for intramural myomas that do not enroach upon the cavity; however, most surgeons would agree to remove them if they are larger than 7 cm or are associated with multiple failed IVF cycles [19]. Only a few reports showed the significant detrimental effect of intramural myoma on ART outcome as detailed in Table 3[5,6,20]. However, meta-analysis results of all reported studies showed the negative effect of fibroid on pregnancy rates (Figure 1, odds ratio 0.737, 95% confidence interval 0.647-0.840, p = 0.000).

**Table 3:** Outcome of ART cycles in women with intramural fibroids and in a control group (without myoma), reporting^**^significant and^*^non-significant results

**Authors**	**Year**	**Type**	**Size (mm)**	**Myoma group**				**Control group**			
				**No. of patients**	**Implantation (%)**	**Pregnancy (%)**	**Abortion (%)**	**No. of patients**	**Implantation (%)**	**Pregnancy (%)**	**Abortion (%)**
***Stovall et al.*** [6]^**^	1998	Prospective IVF/ET and ZIFT^b^	10-54	91 (SS 5, IM 86)	46/334^a^ (13.8%)	34/91^a^ (37.4%)	4/34 (11.8%)	91	65/330^a^ (19.7%)	48/91^a^ (52.7%)	4/48 (8.3%)
***Eldar-Geva et al.*** [5]^**^	1998	Retrospective IVF/ET and GIFT^b^	23.7 ± 7.1	55 IM	NA^a^ (6.4%)	9/55^a^ (16.4%)	3/9 (33.3%)	318	NA^a^ (15.8%)	98/318^a^ (30.8%)	20/98 (20.4%)
***Hart et al.*** [20]^**^	2001	Prospective IVF/ICSI	≤50	86 (IM or SS)	NA^a^ (11.9%)	20/86^a^ (23.3%)	4/20 (20.0%)	290	NA^a^ (20.2%)	99/290^a^ (34.1%)	NA
***Surrey et al.*** [8] **	2001	Retrospective IVF/ET (Age <40 y)	20 ± 2	51 IM	36/168^a^ (21.4%)	27/51 (52.9%)	2/27 (7.4%)	257	265/796^a^ (33.3%)	166/268 (61.9%)	12/166 (7.2%)
***Gianaroli et al.*** [21]^**^	2005	Retrospective IVF/ICSI^b^	18 ± 14	75 IM	48/267^a^ (18.0%)	45/129 (34.9%)	18/45^a^ (40.0%)	127	63/238^a^ (26.5%)	53/129 (41.1%)	10/53^a^ (18.9%)
***Girgin et al.*** [22]^*^	2005	Retrospective IVF/ICSI^b^	30-60	95 IM	NA	26/95^a^ (27.4%)	2/26 (7.7%)	100	NA	43/100^a^ (43.0%)	2/43 (4.7%)
***Khalaf et al.*** [17]^**^	2006	Prospective IVF/ICSI	23 ± 11	112 (IM or SS)	NA	NA^a^ (23.6%)	NA	322	NA	NA^a^ (32.9%)	NA
***Farhi et al.*** [23]^*^	1995	Retrospective IVF/ET^b^	NA	28 IM/SS mix	31/471 (6.6%)	25/86 (29.0%)^b^	10/25 (40.0%)	50	37/317 (11.7%)	32/127 (25.2%)^c^	8/32 (25.0%)
***Ramzy et al.*** [10] *	1998	Retrospective IVF/ICSI ^b^	3.2 ± 1.1	39 (SS 32, IM 12)	16/128 (12.5%)	15/39 (38.5%)	3/15 (20.0%)	367	165/1192 (13.8%)	123/367 (33.5%)	19/123 (15.5%)
***Dietterich et al.*** [11]^*^	2000	Retrospective IVF^b^ (Age >35 y)	10-20	9 (IM or SS)	NA (33.0%)	NA (56.0%)	NA	NA	NA (32.7%)	NA (64.0%)	NA
***Jun et al.*** [12]^*^	2001	Retrospective IVF	< 70	141 (IM or SS or SM)	NA	43/141 (30.5%)	8/43 (18.6%)	406	NA	169/406 (41.6%)	22/169 (13.0%)
***Yarali et al.*** [14]^*^	2002	Retrospective ICSI/ET^b^	50-100	73 IM	18/183 (9.8%)	16/73 (21.9%)	1/16 (6.2%)	324	102/911 (11.2%)	90/324 (27.8%)	6/90 (6.6%)
***Ng et al.*** [13]^*^	2002	Prospective IVF/ET	25-97.5	77 (SS or IM)	22/159 (13.8%)	20/77 (26.0%)	4/20 (20.0%)	312	62/638 (14.4%)	74/312 (23.7%)	6/74 (8.1%)
***Check et al.*** [17]^*^	2002	Prospective IVF/ET^b^	≤ 50	61 (IM 32, SS plus IM 19)	27/208 (13.6%)	21/61 (34.4%)	7/21 (33.3%)	61	41/203 (20.2%)	29/61 (47.5%)	6/29 (20.7%)
***Oliveira et al.*** [17]^*^	2004	Retrospective IVF/ICSI^b^	< 70	130 IM	NA	63/130 (48.5%)	17/63 (27.0%)	245	NA	110/245 (44.9%)	31/110 (28.2%)
***Ballesteros et al.*** [24]^*^	2006	Retrospective IVF	< 50	65 cycle (IM or SS)	NA	NA (20.0%)	NA (46.1%)	366 cycle	NA	NA (23.2%)	NA (29.4%)
***Klatsky et al.*** [25]^*^	2007	Retrospective IVF	28^d^	94 (IM, SS or IM plus SS)	NA (36%)	44/94 (47.0%)	8/44 (15.0%)	275	NA (36%)	149/275 (54.0%)	14/149 (9.0%)
***Bozdag et al.*** [26]^*^	2009	Retrospective ICSI	5-43	61 IM	33/162 (20%)	22/61 (36%)	6/22 (27%)	444	250/1299 (19%)	167/444 (38%)	31/167 (19%)
***Somigliana et al.*** [27]^*^	2011	Prospective IVF/ICSI	50	119 (IM or SS)	NA (17%)	26/119 (22%)	6/28 (21%)	119	NA (11%)	22/119 (19%)	6/22 (27%)

*Abbreviations*: NA not available, SS subserosal, IM intramural, SM submucosal. ^a^Statistically significant difference from controls. ^b^Age matched. ^c^The rates are given as per embryo-transfer. ^d^Average diameter.

In contrast, according to systematic review of the evidence reported by Pritts et al. [3] those women with only intramural fibroids had relative risks of pregnancy, implantation and delivery of 0.94 (95% CI 0.73–1.20), 0.81 (95% CI 0.60–1.09) and 1.01 (95% CI 0.73–1.34), respectively, when compared with their infertile counterparts without fibroids in *in vitro* fertilization cycles [3]. Furthermore Surrey et al. [28] recommended that submucosal leiomyomas and intramural leiomyomas which distort the endometrial cavity would have deleterious effects on implantation and IVF cycle outcome. However, the effect of intramural leiomyomata which do not distort the cavity is much more difficult to evaluate. Conflicting conclusions from various investigators made difficult to recommend routine myomectomy for all intramural lesions before IVF/ICSI cycle [28]. Many retrospective and only a few prospective matched control studies failed to reveal significant detrimental effect of intramural myoma on IVF/ICSI outcome as detailed in Table 3[8-10,12-16]. The failed significant detrimental effect of myoma on reproduction may be explained with the lack of better classification system of myoma. The wide use of new FIGO classification system (PALM-COEIN) for causes of abnormal uterine bleeding and leimyoma subclassification system [29] in the world may help to elucidate the effect of leoimyoma not distorting endometrial cavity on pregnancy and implantation rates.

The effect of intramural fibroids not distorting endometrial cavity was also investigated in oocyte donation cycles in order to rule out the effect of ovarian stimulation on endometrial receptivity as well as the detrimental effect of age on oocyte quality. The authors failed to reveal detrimental effect of myoma on clinical pregnancy and implantation rates, although they reported alteration in the expression pattern of some endometrial genes [30]. The effect of submucosal myoma on endometrial gene expression was also investigated and the recent study reported that submucosal myomas adversely affect reproduction by decreasing endometrial HOX gene expression [4].

The mechanisms by which myomas may affect reproductive outcome are as follows: interference with sperm transport or access (by anatomic distortion of the cervix, enlarging or deforming the endometrial cavity, altering the uterine contractility, and obstructing tubal ostia) or implantation failure (by physically changing the shape of the endometrium, preventing discharge of intrauterine blood or clots, and altering the normal endometrial development (endometrial vascular disturbances, inflammation, ulceration, thinning, and atrophy and altered biochemical environment)) [4,19]. Intramural myomas particularly <7 cm in size may alter myometrial contraction during ET procedure and cause negative effect on implantation, pregnancy and abortion rates following ICSI/ET cycles, even they do not impinge the endometrial cavity.

*Ng et al.*[13] reported that the presence of fibroids resulted in significantly reduced uterine artery pulsatility and resistance index [13]. Based on this study it may be suggested that the blood flow towards the endometrium may be compromised because of drainage of blood towards fibroids. This effect may explain the detrimental effect of myoma on implantation and pregnancy rates in ICSI/ET cycles.

In this retrospective controlled study, the effect of single intramural myoma (<7 cm) not distorting endometrial cavity was investigated and we found that the fibroid group had lower implantation ad pregnancy rates. This may suggest the detrimental effect of myoma on reproduction. As seen in Table 3, the significant detrimental effect of such size myoma on reproduction has not been reported previously. However, the main limitations of this study are the limited size of groups and the retrospective study design. In order to make a certain conclusion regarding intramural fibroids below 7 cm in size not distorting intrauterine cavity, a randomized controlled trial (RCT) of myomectomy versus no therapy should be performed in ICSI/ET cycles. Furthermore, the best study to determine the effects of intramural fibroids on the ART outcome would be a prospective cohort study and then a RCT would best demonstrate any possible benefit of myomectomy to mitigate these potentially detrimental effects.

## Conclusions

Based on our preliminary results of this study, although significant results were obtained in terms of implantation and pregnancy rates, it is not easy to advocate removal of intramural fibroids to improve fertility before ART cycles, since there are many potential complication associated with myomectomy, such as infection, bleeding, possibly blood transfusion, damage to abdominal or pelvic organs, formation of intraabdominal adhesions and anesthesia related complications and possible subsequent pregnancy complications [9]. However, the current study significant results in terms of implantation and pregnancy rates may suggest the possible positive effect of myomectomy on ICSI/ET outcome for only particular myoma size.

## Abbreviations

ART: Assisted reproductive technologies; ICSI: Intra cytoplasmic sperm injection; IVF: In vitro fertilization; PR: Clinical pregnancy rate; AR: Abortion rate; IM: Intramural; SS: Subserosal.

## Competing interests

The authors declare that they have no competing interests.

## Authors’ contributions

SG, CK, and MAU carried out the clinical part of the study and drafted the manuscript. EO carried out the laboratory part of the study and drafted the manuscript. All authors participated in the design of the study and SG performed the statistical analysis. All authors read and approved the final manuscript.
